# Might Gut Microbiota Be a Target for a Personalized Therapeutic Approach in Patients Affected by Atherosclerosis Disease?

**DOI:** 10.3390/jpm13091360

**Published:** 2023-09-07

**Authors:** Marco Matteo Ciccone, Mario Erminio Lepera, Andrea Igoren Guaricci, Cinzia Forleo, Concetta Cafiero, Marica Colella, Raffele Palmirotta, Luigi Santacroce

**Affiliations:** 1 Cardiology Unit, Interdisciplinary Department of Medicine, School of Medicine, University of Bari “Aldo Moro”, 70124 Bari, Italy; marcomatteo.ciccone@uniba.it (M.M.C.); marioerminio.lepera@uniba.it (M.E.L.); andreaigoren.guaricci@uniba.it (A.I.G.); cinzia.forleo@uniba.it (C.F.); 2 Area of Molecular Pathology, Anatomic Pathology Unit, Fabrizio Spaziani Hospital, 03100 Frosinone, Italy; concettacafiero@gmail.com; 3 Interdisciplinary Department of Medicine, Section of Microbiology and Virology, School of Medicine, University of Bari “Aldo Moro”, 70124 Bari, Italy; marycolella98@gmail.com (M.C.); luigi.santacroce@uniba.it (L.S.)

**Keywords:** microbiota, cardiovascular diseases, atherosclerosis, TMAO, probiotics, nutraceuticals

## Abstract

In recent years, the increasing number of studies on the relationship between the gut microbiota and atherosclerosis have led to significant interest in this subject. The gut microbiota, its metabolites (metabolome), such as TMAO, and gut dysbiosis play an important role in the development of atherosclerosis. Furthermore, inflammation, originating from the intestinal tract, adds yet another mechanism by which the human ecosystem is disrupted, resulting in the manifestation of metabolic diseases and, by extension, cardiovascular diseases. The scientific community must understand and elucidate these mechanisms in depth, to gain a better understanding of the relationship between atherosclerosis and the gut microbiome and to promote the development of new therapeutic targets in the coming years. This review aims to present the knowledge acquired so far, to trigger others to further investigate this intriguing topic.

## 1. Introduction

Almost 50% of all deaths in Europe are attributed to cardiovascular diseases. Lifestyle changes, i.e., increased consumption of saturated short-chain fatty acids (SCFAs) and lack of physical exercise, combined with increased life expectancy have shifted the etiology of population mortality to cardiovascular disease and cancer [1,2,3,4,5]. The 2023 World Heart Report reports that more than half a billion people worldwide continue to fall ill from cardiovascular disease, with 20.5 million deaths in 2011 (almost a third of all deaths globally) increasing overall to an estimated 121 million deaths from cardiovascular disease in 2022 [3,4]. Atherosclerosis (from the Greek words *athira*, meaning porridge, and *sklèrosis*, meaning hardening) is a type of arteriosclerosis (from the Greek *artirìa*, meaning artery, and *sklèrosis*), which refers to loss of elasticity of the arteries. This hardening occurs due to the accumulation of atheromatous plaque. The term atherogenic refers to the substances or processes through which atheroma is created. Atherosclerosis is the leading cause of vascular disease worldwide. It is a multifactorial disease that is influenced by both hereditary and environmental factors [1,2]. Cardiovascular disease mortality is higher in underdeveloped and developing countries than in developed countries. Furthermore, atherosclerosis describes the disease of the arteries, characterized by inflammation and dysfunction of the endothelium, as well as the accumulation of lipids, cholesterol, calcium, macrophages, and smooth muscle cells in the intima of the vessel. It is one of the leading causes of mortality and morbidity in the Western world. Thus, cardiovascular diseases such as heart attack, peripheral arterial disease, and others are the clinical manifestations of atherosclerosis [6]. Dyslipidaemia, diabetes mellitus, arterial hypertension, smoking, psychosocial factors, obesity, limited physical activity, and family history are the main risk factors for atherosclerosis. Newer risk factors include increased markers of inflammation (C-reactive protein), hyperhomocysteinemia, renal disease, endothelial dysfunction, and hypertriglyceridemia. Other factors that indirectly affect the promotion of atherosclerosis are sleep apnoea, stress, and alcohol [7].

The atheromatous plaque is formed by the complex deposition of intra- and extracellular substances in the intima, constituting the patho-anatomical substrate of atherosclerotic coronary disease. Atherosclerotic plaques are characterized by various forms and stages of progression and may vary significantly in morphology, even within the same patient. Early lesions are characterized by the infiltration of foamy cells, which are macrophages containing lipid oxidation products and cholesterol esters (type I). As these lesions mature, they are infiltrated by smooth muscle cells and other lipids, while foamy cells increase and are organized into layers (type II—fatty streaks). This is followed by connective tissue deposition and accumulation of extracellular lipids (type III—proatheroma) [8,9]. These early lesions usually develop during the first three decades of life at sites of coronary arteries with local turbulent flow. Their development is accelerated by coexisting conditions, such as arterial hypertension, diabetes mellitus, hyperlipidaemia, smoking, and other cardiovascular risk factors. The type IV lesion (atheroma) contains a large amount of extracellular lipids and occupies an extensive but well-circumscribed area of the intima. The part of the lesion occupied by these lipids is known as the “fatty nucleus” (or fatty core), which is formed by the growth and subsequent fusion of small and isolated aggregates of extracellular lipids, which characterize type III lesions [9]. The accumulation of lipids is thought to be due to their continuous entry from the plasma into the interior of the lesion. In contrast to early lesions, the type IV lesion is the first lesion to be considered advanced, due to the significant disruption caused by the fatty core to the intimal structure. In type IV lesions, the intima, in addition to cellular infiltration, does not show significant collagen and extracellular substance infiltration. When an increase in the deposition of collagen fibrils and formation of a fibrous tissue layer at the boundaries of the fatty core (fibrous capsule) occurs, the lesion is characterized as type V (fibro-atheroma) [9,10,11]. Type IV lesions also appear in younger individuals as eccentric thickenings of the intima. Thus, at least initially, atheroma is an eccentric lesion. Often, atheroma does not cause narrowing of the lumen of the coronary artery; on the contrary, it can lead to an increase in the outer limits of the vessel (positive remodelling). The tissue interposed between the fatty core and the vascular lumen contains macrophages and smooth muscle cells, with or without fat droplets, lymphocytes, mast cells, and abundant capillaries, while the density of these elements is greater at the periphery of the lesion. The formation of the fatty core precedes the development of fibrous tissue, which gradually change the characteristics of the intima overlying the fatty core. Type V lesions are subdivided into type Va lesions (when the fibrous capsule surrounds a clearly delineated fatty core), type Vb lesions (when they are calcified by the fatty core or other parts of the lesion) and in type Vc lesions (when the fatty core is barely distinguishable or indistinguishable due to calcification). These lesions cause strictures of various sizes, which are generally larger than the strictures caused by type IV lesions. 

The clinical significance of type IV lesions is great, although these advanced lesions do not always cause significant narrowing of the vascular lumen. Because the area between the fatty core and the intima contains abundant proteoglycans, many foam cells, and few individual smooth muscle cells with little collagen, this damage often leads to intimal breakdown and formation of fissures and ulcerations; this is the type VI injury. Thus, the peripheral area of type IV lesions is particularly vulnerable to rupture, due to the abundance of macrophages in these areas. Lesions with simple discontinuity of the overlying intima are called type VIa, those with hematoma or haemorrhage are type VIb, and ones with thrombus are type VIc. The lesion is categorized as the type VIabc classification when all three of these elements are present [12,13]. The disruption of the intimal continuity is facilitated by the presence of inflammatory cells, the release of toxic substances and proteolytic enzymes from the macrophages located within the lesion, the spasm of the coronary vessel, the structural weakness around the lesion associated with plate composition, and the increased shear stress. The cracks are more often found in areas where there is a large concentration of macrophage–foamy cells, while sometimes, during their healing, they incorporate hematomas and thrombi within the lesion [14]. Although intimal hematomas are usually caused by tears in the surface of the plaque, there is evidence that some hematomas may initiate within the lesions as a result of microscopic ruptures of newly formed blood vessels. The creation of cracks and hematomas is usually accompanied by the formation of clots of various size. Large occlusive or non-occlusive thrombi usually lead to acute coronary events, while small thrombi do not always cause symptoms [15]. Inflammation appears to mediate the initiation and the progression of the atherosclerotic process, as it contributes to the increase in endothelial permeability, leukocyte adhesion and infiltration, and lipid accumulation in the arterial intima [16]. Thus, inflammation is a common denominator of a series of cardiovascular risk factors, such as hyperlipidaemia, hypertension, smoking, etc. [17,18]. In these conditions, the endothelial cells begin to locally express adhesion molecules on their surface, which bind to certain classes of white blood cells. Vascular cell adhesion molecule-1 (VCAM-1) binds to monocytes and T-lymphocytes, which are detected in early-stage atheromatous plaques. The site of maximum expression of adhesion molecules coincides with those parts of the arterial wall which are prone to the development of atheroma. Also, turbulent flow can lead to increased production of certain white blood cell adhesion molecules (e.g., intercellular adhesion molecule-1 (ICAM-1)). Increased wall tension may promote the production of proteoglycans by arterial smooth muscle cells. These proteoglycans can bind and retain lipoprotein particles, facilitating their oxidative modification and, thus, promoting inflammatory response at the sites of atherosclerotic damage [19]. Immediately after their attachment to the endothelium, white blood cells infiltrate the intima, where they participate in and facilitate the local inflammatory response. Macrophages, in response to the inflammatory process, express receptors for modified lipoproteins, which allow the macrophages to assimilate lipids and, thus, transform into foamy cells. Likewise, T cells are stimulated to produce inflammatory cytokines, such as interferon and tumour necrosis factor (TNF), which, in turn, activate macrophages and endothelial and smooth muscle cells [20]. As this inflammatory process continues, activated white blood cells and other cells release a series of factors that promote smooth muscle cell proliferation and the synthesis of extracellular matrix components. Inflammatory processes not only promote the formation and progression of atherosclerotic plaque, but also contribute decisively to causing erosion, rupture, or ulceration and thrombosis of the atherosclerotic plaque, with the ultimate consequence of the appearance of various clinical forms of coronary disease [21]. Inflammation contributes to the rupture of the atheromatous plaque, as it helps to thin the fibrous capsule due to excessive stimulation of macrophages, causing release of metalloproteinases and other proteolytic enzymes from them. Finally, mobilization and enhancement of inflammatory mechanisms that contribute to atherogenesis have been attributed to some infections (especially herpetic and chlamydial infections) and, in certain cases (such as diabetes, immunodepression etc.), may can lead to sepsis and the rapid multi-organ failure (MOF) condition. However, regardless of aetiology, the role of infection and especially inflammation has been the subject of intensive research [22,23,24].

## 2. The Gut Microbiota

The entirety of the genetic material of the microorganisms that colonize the body constitutes the microbiome, and microbiota represents the combination of several microorganisms such as bacteria, viruses, and fungi in a singular environment. The human intestinal tract hosts a complex and dynamic population of microorganisms—the intestinal microbiota—which plays an important role in the homeostasis of the organism and its diseases. The evolution of the gut microbiota occurs as a result of several factors over the course of life. Diet is considered one of the main factors in shaping the composition of the intestinal microbiota during human life. Intestinal bacteria hold a prominent position in maintaining the body’s defences, homeostasis, and protection against various pathogens. The modified composition of the intestinal microbiota (intestinal dysbiosis), i.e., any deviation from the normal one, has been associated with the pathogenesis of numerous inflammatory diseases and infections [25]. The human gastrointestinal tract consists of the largest surface in the human body (250–400 m^2^). Over one’s lifespan, around 60 tons of food pass through the gastrointestinal tract, along with a variety of microorganisms from the environment which pose a potential threat to intestinal integrity [26]. The collection of bacteria, archaea, and eukaryotes that colonize the gastrointestinal tract is defined as the “gut microbiota” and has evolved with the host over centuries of existence. The number of microorganisms that colonize the gastrointestinal tract is estimated to exceed 10^14^, which means that there are 10 times more than the number of human cells and 100 times more in the microbiome than the genome [27]. However, a recent estimate suggests that the ratio of human/bacterial cells is about 1:1. The sheer number of microorganisms and human cells results in a super-organism. The gut microbiota provides many benefits to the body through a variety of physiological functions, such as enhancing intestinal integrity or shaping the intestinal epithelium, protecting against pathogens, conserving energy, and modulating the immune system. However, there is a possibility that these mechanisms may be disrupted or may malfunction, resulting in intestinal dysbiosis [28]. The gut microbiota has been associated with many enteric and non-intestinal diseases due to this interaction with the host [29,30]. A decade ago, the information we had about the gut microbiome came only from laboratory cultures. Nowadays, the ability to study the gut microbiome has clearly improved due to approaches that do not depend on cultures, such as sequencing methods that are quite economical and make a decisive contribution to this field. A popular approach is to target the 16S ribosomal RNA (rRNA) gene, as this is present in all bacteria and archaea and is, thus, easy to distinguish between different species [31]. Also, reliable estimates of gut microbiota composition can be provided by whole genome metagenomics of microorganisms, due to the greater sensitivity of these techniques. After studies, around 2172 species of microorganisms were identified, which were isolated from the human body and categorized into 12 different classes, of which 93.5% belonged to *Pseudomonadota, Bacillota, Actinomycetota*, and *Bacteroidota* [32]. In humans, 386 of the identified species were strictly anaerobic and were, therefore, found in mucosal regions such as the oral cavity and gastrointestinal tract. An extensive catalogue of the functional capacity of the human gut microbiota has been established, in which 9,879,896 genes have been identified. The composition of the gut microbiota is influenced by many environmental factors such as diet and interaction with the host genome [32,33]. Nevertheless, gut microbiota that differ in composition may share many common features, such as shared proteins or metabolic profiles. This information is useful for the development of therapeutic strategies aimed at modifying the microbiota to combat disease [34].

## 3. Development of the Gut Microbiota

The assumption that the development of the gut microbiota begins at birth has been challenged by a limited number of studies in which the microbiome could be detected even in the placenta of pregnant women. After birth, the gastrointestinal tract is rapidly colonized by the intestinal microbiota. Some life events such as various diseases, antibiotics, and dietary changes cause chaotic changes in the newborn microbiota [35]. Furthermore, the microbiota is transferred from the cervix during normal childbirth, with a greater presence of the *Lactobacillaceae* family, which abounds in the cervical microbiota. In contrast, in newborns born by caesarean section, the microbiota is weakened and slow to be colonized by the genus *Bacteroides*; instead, mainly opportunistic pathogens such as those from *Clostridiaceae* family are found [28]. Newborns born by normal delivery carry 72% of their mother’s microbiota, while those born by C-section carry 41%. In the early stages, the gut microbiota has lower diversity, consisting mainly of *Acinetobacteria* and *Proteobacteria* [34,35,36]. During the second year of life, diversity increases and begins to take on an adult configuration, with some unique individual patterns [37]. Although in adulthood the composition of the microbiota is relatively stable, it is still vulnerable to various life events that may modify it. In people over age 65, the composition changes, with a predominance of *Bacteroides* and *Clostridiaceae* family (such as *Clostridia* class IV); in contrast, in younger individuals, class XIVa is predominant [38]. Overall, the ability of the microbiota to carry out metabolic processes such as the production of SCFAs and the lysis of amyloid decreases; instead, the proteolytic activity increases. Given the increased importance of SCFAs as metabolic and immune mediators, it appears that their depletion may enhance the age-related inflammatory process in the intestinal tract of elderly humans [39].

## 4. Gut Microbiota as an Endocrine Organ

Unlike other endocrine organs or systems that secrete one or a small number of chemical compounds, the gut microbiota has the advantage of producing hundreds of such compounds and products. From a morphological and biochemical point of view, it is vastly larger and more biochemically heterogeneous than any other endocrine organ in the human body. In fact, the biochemical complexity of the gut microbiota exceeds even that of the human brain, and many of the hormones produced by the gut microbiota are also neurotransmitters in the central nervous system (CNS) [40]. For example, γ-aminobutyric acid (GABA), which is one of the most important inhibitory neurotransmitters in the CNS, is produced by various species of the Lactobacillaceae family, while various monoamines, such as noradrenaline, dopamine, and serotonin, are produced by various bacterial strains within the microbiota [41]. This biochemical prowess of the microbiome comes from the large content and diversity of microbial cells, with an estimated mass of 1–2 kg in an average adult. The number of cells is much greater than the number of host cells, and it is estimated that 90% of the cells found in the human body are essentially prokaryotic cells that comprise over 35,000 bacterial species from approximately 1800 genera. Therefore, the diversity of this endocrine system is enormous, and, in adults, about 8 million genes correspond to a small number of genera of microorganisms. Thus, our estimate of the functional repertoire of the gut microbiota is approximate and based on laboratory culture techniques, but most intestinal bacteria are not culturable in the laboratory. A better understanding of the various functions of the gut microbiota is essential to harness it as an endocrine system [42].

## 5. Gut Microbiota Dysbiosis and Atherosclerosis

**Figure 1:**
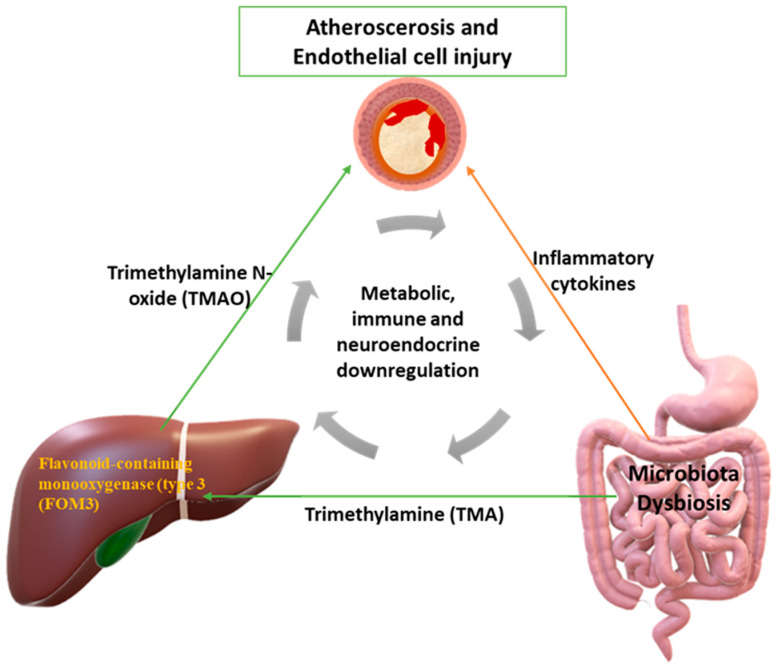
Initially, trimethylamine (TMA) is produced by the microbiota in which, under dysbiosis, bacteria prevail, such as those of *Prevotella*-containing flavonoids (type 3 (FOM-3)), leading to an increase in TMAO production. This increase in the production of TMAO fosters the formation of atheroma, causing local alterations, sclerosis, and damage to the endothelial structure and function. Thereafter, dysbiosis and intestinal and peripheral inflammation (such as inflammation of the vessels) leads to more and more immune downregulation and metabolic neuroendocrine, aggravating the state of cardiovascular health.

The intestinal microbiota plays an important role in the human immune system and takes part in its development. Intestinal dysbiosis may promote atherosclerosis by modulating the immune system. Mediators of the microbiome in this direction are NOD-like receptors (NLR), myeloid differentiation factor 88 (MyD88), and Toll-like receptors (TLRs), including TLR4, TLR3, and TLR2. The latter are directly linked to the development of atherosclerosis [43]. Platelets are activated by downstream signaling pathways of these receptors (myeloid differentiation factor 88 (MyD88) and Toll-like receptors (TLRs), including TLR4, TLR3 and TLR2), specifically as damage-associated molecular patterns (DAMPs). Receptors involved in platelet activation have been found before [44]. Thus, this process may highlight a key role of the gut microbiota in underlying platelet activation in patients with CAD. A high-calorie diet with increased SCFAs affects the permeability of the intestinal wall, modifying the composition of the intestinal microbiota and the “tight” junctions (ZO-1) between the cells of the intestinal wall. This results in bacterial fragments and uremic toxins being transported into the bloodstream, either through extracellular leakage between junctions or through transport by a chylomicron-mediated mechanism [45,46]. In particular, the connections between the cells of the intestinal wall are affected by the production of nitrite components (ammonia and ammonium hydroxide) which come from intestinal dysbiosis (the microbiota hydrolyses urea and, thus, these chemicals are produced) [47]. Some of these fragments are lipopolysaccharides (LPS) and glycolipids that lead to endotoxemia, thereby activating macrophages and NK cells [48]. In addition, saturated SCFAs are associated with the differentiated composition of the intestinal microbiome and are ligands for TLR4 receptors [49]. When binding to the TLR4/CD14 receptors in macrophages, they induce a cascade of pro-inflammatory cytokines (mainly IL-6 and TNF-a), thereby initiating an immune response. As previously mentioned, inflammation is a key risk factor for atherosclerosis. Studies have found DNA from the microbiota in the arterial plaque, mainly in combination with the overexpression of the genus Proteobacteria [50,51]. DNA levels correlated with leukocyte and B2 cell levels in atherosclerotic plaque and appeared to influence plaque stability [52]. Furthermore, it has been reported that metabolic products from the intestinal microbiota activate B2 cells and their production of IgG, which affects the promotion of atherosclerosis. This has been demonstrated, as atheromatous plaque generation was considerably decreased in patients receiving antibiotics that reduced these metabolic products [53,54,55]. Diet is the main factor that determines the composition of the microbiota. The diversity of the composition of different microbial colonies contributes to and is associated with various metabolic disorders such as atherosclerosis. Low levels of Bacteroidota and intestinal dysbiosis of opportunistic pathogens such as Enterobacter, Collinsella, Desulfovibrio, and Klebsiella genera are factors associated with atherogenesis and atherosclerotic plaques [29,46,56] Furthermore, apart from their absolute number, the relative ratio between them is also of great importance, which also plays a role in metabolic diseases. It has been reported that Bacteroidota play an important role in regulating the actions of T cells and, by extension, the body′s immune system [46,56,57,58,59]. In addition, infections by Helicobacter pylori are associated with an increased atherogenic profile, due to lipopolysaccharide (LPS), which characterizes this genus of microbes and their ability to cause vascular inflammation. This occurs when LPS passes into systemic circulation through the intestinal wall. As mentioned above, variations in the composition of the intestinal microbiome enhance the production of TMA, the precursor to TMAO. Finally, there are many studies linking intestinal dysbiosis to specific classes of lipids only, such as HDL and VLDL. In the intestinal tract, trimethylamine N-oxide (TMAO) is produced by the metabolism of L-carnitine, choline, betaine, and phosphatidylcholine. Initially, trimethylamine (TMA) is produced by the intestinal microbiome. In the liver, trimethylamine (TMA) is converted to trimethylamine N-oxide (TMAO) by flavonoid-containing monooxygenase type 3 (FOM-3) (Figure 1). Choline is converted to trimethylamine (TMA) via CutC and CutD, which are the genes responsible for the formation of trimethylamine (TMA) from choline via choline TMA-lyase [60]. Also, trimethylamine N-oxide (TMAO) is produced, as mentioned above, from L-carnitine, through two different pathways. The exact relationship of how TMAO is associated with atherosclerosis is not yet known. Several studies remain to be carried out in this direction. The first pathway is through the direct conversion of L-carnitine to TMAO by the gut microbiota, and the second pathway is through the conversion of L-carnitine to γBB (γ-butyrobetaine), a process that takes place in the small intestine, which is then converted to TMA and then to TMAO via FOM-3. In patients with atherosclerotic disease in their carotid arteries, it was found that the levels of γ-butyrobetaine (γ-BB) and L-carnitine were higher than those in the healthy group. Of note, γ-butyrobetaine is also produced from trimethyllysine (TML), independently of L-carnitine. Furthermore, TML and γ-BB have been found to be associated with atherosclerosis, independently of TMAO production (i.e., their indirect involvement in the promotion of atherosclerosis via TMAO) [61,62]. Regarding the silencing of genes which produce FOM-3, this induces a syndrome in patients, who then emit a fishy odor due to trimethylaminuria production [63,64]. The reason for this unpleasant odor is that TMA is excreted by the kidneys, sweat, and breath. Foods that are rich in TMAO are mainly red meat, egg yolks, and sea fishes. Nevertheless, fish is a key element in a balanced, cardioprotective diet. This beneficial effect, from eating fish, probably comes from the omega-3 fatty acids. Thus, the intake of TMAO from eating fish is likely to be proportionally negligible in relation to the benefits of eating it [65]. It has also been found in studies that vegetarians have lower levels of TMAO than those who also consume meat [66,67,68,69]. Elevated L-carnitine levels combined with elevated TMAO levels were found to have a combined role in increased cardiovascular risk (myocardial infarction, stroke, and death). In addition, the content of the intestinal microbiota also contributes to determining the levels of TMAO in the bloodstream. This is because different microbes have varying ability to produce TMAO. For example, Prevotella species are more prone to TMAO production than Bacteroides spp. Also, it has been reported that vulnerability to atherosclerosis can also be promoted by fecal transplantation [70,71]. Trimethylamine N-oxide (TMAO) suppresses cholesterol efflux by blocking its reverse transport to the liver; this is yet another way by which TMAO promotes atherosclerosis (Figure 1). Bile acid concentration is altered and reduced due to TMAO, as well as its synthesis and secretion (there is reduced activity of FXR (farsenoid X receptor) signaling—this type of signaling has to do with bile acids and affects lipid metabolism) [72,73,74,75]. Some other effects are the reduction of cholesterol absorption from the enterocyte, increasing glucose tolerance and promoting adipose tissue inflammation. It is evident that all these effects indirectly affect the development of atherosclerosis [76,77]. Additionally, TMAO is associated with higher-level syntax levels. Many authors have considered whether TMAO is a clinical marker of atherosclerosis or is the cause of atherosclerosis. For example, if there is atherosclerosis in the renal arteries with concomitant atherosclerosis in the coronary arteries, then TMAO would be a marker for renal dysfunction [78]. TMAO also increases macrophage scavenger receptors, which results in macrophage cholesterol accumulation and foamy cell formation [79,80,81,82]. Another pathway by which TMAO contributes to the development of atherosclerosis and, by extension, to cardiovascular disease is through inducing platelet hyperreactivity. This is due to the change in the calcium levels in the platelets. This increases the risk of thrombosis [82,83]. TMAO can increase platelet activation through a direct interaction or it can indirectly affect platelets via the elevation of cytokine/chemokine released from other cells such as leukocytes, which interact with platelets in indirect ways [83,84]. TMAO is also responsible for the inflammation of vascular endothelial cells because it induces the chemotaxis of leukocytes, increases the expression of pro-inflammatory cytokines, and promotes the secretion of IL-6, E-selectin, and adhesion molecules (in essence, it promotes the transcription of the corresponding genes of such substances) [44,85]. It also inhibits endothelial nitric oxide synthase (eNOS) and the bioavailability of nitric oxide (NO), instead activating the products of oxidative stress (ROS), which lead to the inflammation of endothelial cells. In turn, ROS can activate platelets, thus enhancing their proinflammatory phenotype, complicating atherosclerosis [85,86,87,88,89]. High levels of TMAO are associated with hypomethylation conditions and a specific pattern of the lipid profile, in which phospholipids and HDL are detected at low levels. Male gender may also play a role in TMAO production [90]. TMAO has been found to be a potential clinical marker in patients with peripheric vasculopathy. In rats, the Slc30a7 gene has been reported to play an important role in TMAO production and levels, but, in humans, the mechanism appears to be much more complex than one specific gene being involved [91].

## 6. Potential Therapeutic Targets

**Figure 2:**
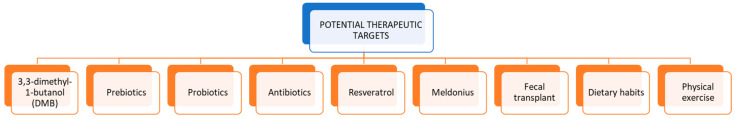
Main therapeutic approaches to prevent the development and promotion of atherosclerosis through “modulation” of the gut microbiota.

Nowadays, there are many potential therapeutic approaches that are used to prevent the development and promotion of atherosclerosis through the gut microbiota (Figure 2). Some are used as modifiers of the intestinal microbiota composition, thus preventing intestinal dysbiosis. Such approaches include antibiotics, probiotics, prebiotics, resveratrol, meldonium, antibiotics, dietary modification (e.g., reduced choline intake and high-fiber diet), fecal transplantation, and physical exercise. Another possible treatment is 3,3-dimethyl-1-butanol (DMB), which is promising [92,93,94,95,96]. 

Initially, during treatment with antibiotics (mainly ampicillin and azithromycin), the composition of the microbiota improved significantly; however, there were several side effects due to the prolonged use of these drugs [97]. Prebiotics have a positive effect on the composition of the intestinal microbiome and promote the growth of symbiotic bacteria in the intestinal lumen. They also cause a reduction in intestinal pathogens that have a negative effect on metabolism and, by extension, a promotion in atherosclerosis. Some prebiotics are oligosaccharides and inulin. Probiotics are known as live microorganisms with a positive effect on the human body in the same way as prebiotics. Some of them are *Bifidobacteria, Enterococcus*, and *Lactobacillus* [98,99]. Archaebiotics, such as *Methanomassiliicoccus luminyensis* B10, reduce the production of TMA by reducing methanogenesis in the intestinal lumen, which is a therapeutic target for atherosclerosis [97]. Regarding dietary food intake, resveratrol (a natural phytoalexin) behaves as a prebiotic and increases the proportion of *Bifidobacteria* and *Lactobacillus* [100,101]. Plant sterol ester (PSE) and β-glucan (OBG) have been reported as dietary interventions that reduce the promotion of atherosclerosis by the gut microbiome through the reduction of TMA levels [102,103]. Furthermore, SCFAs such as butyric acid and pomegranate have an anti-inflammatory effect, reducing pro-inflammatory cytokines. This results in a reduction in atherosclerotic plaque [104,105]. In addition to butyric acid, some probiotic products, such as berberine, kefir, and Greek yogurt, are reported in the international literature for their anti-atherosclerotic role by modifying the composition of the microbiota [106,107]. Meldonium, an analogue of γ-butyrobetaine, was found to reduce TMA production [108]. Other dietary habits that may be beneficial are red berries, tea polyphenols, and oligosaccharide mannans [109,110]. Beyond affecting the composition of the microbiota, many therapeutic approaches interfere with TMAO production. DMB, which is a choline analogue, inhibits TMA lyases and, consequently, the production of TMA by the gut microbiome, thereby reducing TMAO levels [111]. DMB is found in virgin olive oil and wine [112]. In addition, DMB inhibits the generation of foamy cells and, thus, reduces the promotion of atherosclerosis [113,114,115,116]. The FOM-3 enzyme has many positive effects on the human body and, for this reason, its suppression has many side effects such as liver inflammation [117,118]. In fact, when there is a genetic disorder in the function of FOM-3, patients suffer from fish odor syndrome, which is one of the side effects of suppressing this enzyme [117,118]. Thus, the above therapeutic path is not an attractive intervention, despite the initial enthusiasm. Finally, bariatric surgery did not appear to decrease TMAO levels, even though some metabolic parameters improved [119].

## 7. Conclusions

By studying and understanding the pathophysiology of the promotion of atherosclerosis by the dysbiosis of the gut microbiota, we lay the foundation for the further search for new therapeutic targets. For this reason, more evidence-based studies, based on a large statistical sample, are needed, both to understand the exact pathophysiology and to test new chemical therapeutic targets through large multicentered studies, initially in experimental animals, and later in humans.

## Data Availability

Data sharing is not applicable.
